# Enamel Roughness Changes after Removal of Orthodontic Adhesive

**DOI:** 10.3390/dj6030039

**Published:** 2018-08-06

**Authors:** Felipe Weidenbach Degrazia, Bruna Genari, Vilmar Antonio Ferrazzo, Ary dos Santos-Pinto, Renésio Armindo Grehs

**Affiliations:** 1 Department of Orthodontics and Biomaterials, Centro Universitário UDF, Brasília, DF 70390-045, Brazil; bruna.genari@gmail.com or bruna.genari@udf.edu.br; 2 Department of Orthodontics and Craniofacial Orthopedics, Universidade Federal de Santa Maria, Santa Maria, RS 97015-372, Brazil; vilmarferrazzo@uol.com.br (V.A.F.); renesiogrehs@smail.ufsm.br (R.A.G.); 3 Department of Children’s Clinic—Orthodontics, Universidade Estadual Paulista, Araraquara, SP 14801-903, Brazil; aryspinto@gmail.com

**Keywords:** dental enamel, orthodontic brackets, dental bonding, dental debonding

## Abstract

The aim of this study was to evaluate enamel roughness, quality of the enamel surfaces and time duration comparing different orthodontic adhesive removal protocols. Premolars were used to test three adhesive removal methods (*n* = 20): five-blade carbide bur, 30-blade carbide bur, and ultrasonic diamond bur. Bracket was bonded using Transbond^TM^ XT adhesive. Roughness with different parameters was measured before bracket bonding and after adhesive remnants removal. Micromorphological analysis of enamel surface (*n* = 5) was performed by SEM images and categorized in enamel damage index—“perfect”; “satisfying”; “imperfect”; and “unacceptable”. Time was measured in seconds. All removal methods caused increased roughness in relation to *R*_a_, *R*_q_, and *R*_z_ parameters (*X* axis) comparing to healthy enamel surface. Enamel surface resulted from removal using five-blade burs was scored as satisfactory. Carbide bur groups decreased the roughness values of *R*_a_, *R*_q_, and *R*_z_ parameters on the *Y* axis and enamel surface was considered unacceptable. The 30-blade group increased symmetry (*R*_sk_) and flattening (*R*_ku_) parameters of roughness and surface was scored as unsatisfactory. Diamond bur removed adhesive in 54.8 s, faster than five-blade carbide bur. The five-blade bur group resulted in less enamel roughness than the 30-blade and diamond groups.

## 1. Introduction

The restoration of the original structure of enamel, which is found before orthodontic treatment with fixed appliances, may be achieved after the total removal of the remaining post-debracketing adhesive [1]. This removal process is a challenge since it often affects the enamel surface, leading to an increase in roughness, enamel cracking and tarnishing from lingering residue [2,3]. It also leads to anatomical alterations with consequent bacterial retention [4] and optical property changes, such as light reflection of the enamel crystals [5]. In an epidemiological survey of debonding techniques, 36% of orthodontists reported enamel surface damages after debonding [6].

In order to minimize the damage of enamel surface during the bracket-removal procedures, debonding of the remnant adhesive layer has been tested with different methods in different ways [7,8,9,10,11,12]. The closest results to the initial condition of the enamel were achieved with tungsten carbide burs [3,13,14] and composite burs; however, these burs proved to be inefficient due to a considerable increase of clinical removal time [8]. The use of diamond burs, contraindicated up until now for this removal procedure [14,15], could serve as an alternative to removal with a manufacturing model by chemical vapor deposition. This technology makes diamond particles smaller, around 30.0 µm in diameter size, and distributed evenly when compared to conventional diamond burs. According to a previous study [16], burs with ultrasonic technology have higher wear resistance. Furthermore, they function by vibrating, and they are less noisy.

Enamel roughness has been measured with the aid of devices such as roughmeters, optical or contact profilometers, and atomic force microscopes (AFM) to determine different roughness parameters [8,10,17]. The most commonly applied roughness parameter is general average (*R*_a_); nevertheless, other parameters such as height (*R*_z_), symmetry (*R*_sk_), and flattening (*R*_ku_) define important details about the roughened surfaces [3].

The aim of this study was to evaluate enamel roughness, quality of the enamel surfaces and time duration comparing different protocols for removing orthodontic adhesive: five-blade and 30-blade carbide burs and ultrasonic diamond bur by chemical vapor deposition (CVD).

## 2. Results

The mean values of roughness parameters are summarized in Table 1 and Table 2. Wilcoxon signed-rank test found significant differences on both axes of the tested groups (*p* < 0.001). A significant increase of the parameters *R*_a_ and *R*_q_ were found on the *X*-axis for the 30-bladed carbide and the CVD groups; on the *Y*-axis, the carbide burs groups had significantly decreased the mean roughness scores of *R*_a_, *R*_q_, and *R*_z_ when compared to the initial condition of the enamel surface. The *R*_ku_ and *R*_sk_ parameters were altered only by the carbide bur groups.

The Kruskal-Wallis Multiple Comparisons test revealed statistically significant differences of the roughness parameters on the *X*- and *Y*-axes. The Dunn test showed that the three groups had significant differences of *R*_a_ and *R*_q_ values; an increase occurred on *X*-axis of the roughness parameter *R*_z_ comparing CVD to carbide burs groups, and no significant differences have been found in the parameters *R*_ku_ and *R*_sk_.

Statistical difference among groups (*p* < 0.001) were found at all parameters evaluated on the *Y*-axis. The *R*_q_ and *R*_z_ parameters had significant increase (*p* < 0.001) by the five-bladed and CVD groups when compared to the 30-blade carbide group. The CVD group increased significantly *R*_a_ (*p* < 0.001) comparing to the carbide burs groups, and had significantly decreased *R*_ku_ and *R*_sk_ when compared to carbide burs groups.

**Figure 1:**
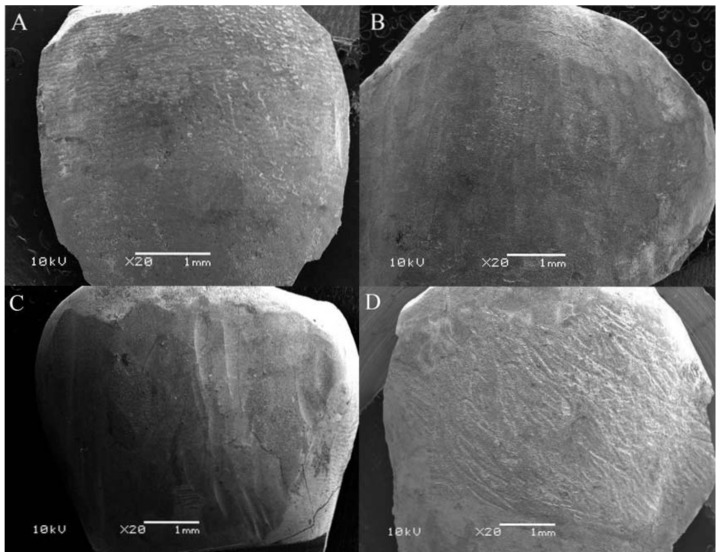
SEM images. (**A**) Healthy Group. (**B**) Group 1 classified as satisfactory score. (**C**) Group 2 showing imperfect surface. (**D**) Group 3 with unacceptable surface.

**Figure 2:**
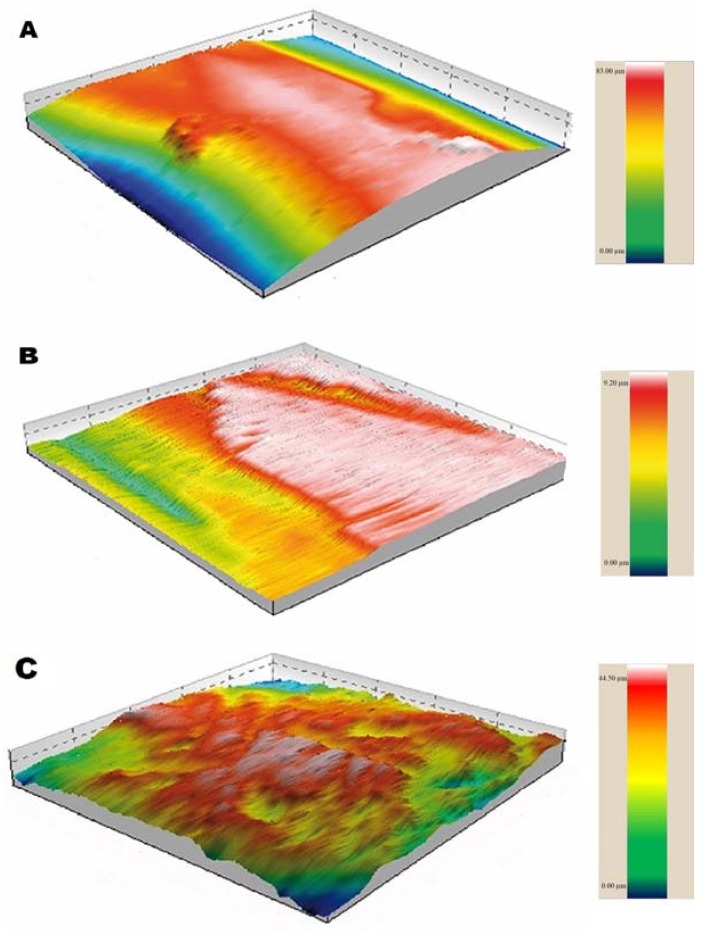
Profilometry images. (**A**) The five-bladed group exhibits a curve profile with higher polishing. (**B**) The 30-bladed tungsten carbide group showed a flat surface with abrupt changes. (**C**) The diamond group (CVD) showed greater amount of pores and irregular contour.

Scanning Electron Microscopy (SEM) images of each group were classified according to Figure 1 and are describe in Table 3. Computerized images of the enamel profile for each group are shown in Figure 2.

Regarding the time required for removal of the remaining resin, summarized in Table 4, there was a significant decrease (*p* < 0.05) from the CVD group compared to the five-bladed carbide group.

## 3. Discussion

The adhesive procedure for removing adhesive remnant remains a concern in orthodontics because rougher surfaces facilitate the retention of bacterial plaque and extrinsic stains [18,19]. Sfondrini et al. [6] recently led an epidemiological survey of different clinical techniques for adhesive removal, showing more than 54% of orthodontists using tungsten carbide burs whereas 36% of them observed enamel changes after orthodontic treatment.

The main finding of our study was to shed light on how the enamel changes after applying different methodologies to remove remnant adhesive. Despite the similar Enamel Damage Index (EDI) scores of healthy teeth and enamel treated with five-blade carbide groups—as also previously demonstrated by Radlanski (2001) [13]—the enamel surface suffered roughness alterations after removal procedures with the five-blade bur. Radlanski claims that few changes in roughness caused by this material are due to the shape of the bur, which has a groove rather than a sharp angle, thus protecting the enamel from possible risks. Our research matches Radlanski’s claim, since there was an increase of roughness caused by the five-blade carbide bur only in the *R*_q_ and *R*_z_ parameters on the *Y*-axis compared to the 30-blade group, and in the *R*_ku_ parameter on the *Y*-axis compared to CVD group. In addition, the five-blade carbide bur presented a smooth contour with less adhesive remnants compared to the other groups (Figure 1). The group of the five-blade carbide bur presented the lowest means of roughness parameters on the *X*-axis, which is consistent with previous researches [13,20]. The decrease in *Y*-axis roughness values, as with the 30-blade group, was unexpected, and may be explained by the perpendicular direction of the cutting blades at the scanned area purporting a polished surface, and suggesting less plaque accumulation [4]. Furthermore, removal of the enamel layer generates excessive polishing, because the enamel deposition lines run through the mesial-distal way of the sample, and the scanning tip runs perpendicular to them [21]. SEM images of the group of the 5-blade carbide bur was classified as satisfactory, since it showed a very similar aspect to the initial images of healthy enamel, despite a previous report of a wavy pattern [13]. These results suggest the adhesive removal using a five-blade carbide bur can be an alternative procedure to promoting reliable roughness.

The removal of adhesive remnants with diamond burs had been inadequate [22,23] for orthodontics; however, with the development of a CVD-applied diamond bur, a new field that uses diamond tools for the removal of orthodontic resin on the enamel could be established. Nevertheless, the following results showed an increase in the average values of *R*_a_, *R*_q_, and *R*_z_ parameters on both the *X* and *Y*-axes compared to the initial analysis. This result was similar to other studies using conventional diamond burs [3,17,22]. Compared to the carbide groups, a greater number of valleys on the *Y*-axis—represented by negative *R*_sk_—and lower roughness values were obtained in the parameter *R*_ku_, showing more obtuse peaks and valleys. This is not necessarily an advantage, since valleys with greater angles might encourage greater deposition of microorganisms, as Quirynen (1994) [4] found with the increase of *R*_a_ parameter assessment. The images of this group, scored as unacceptable, detract comparing to a healthy condition; no presence of the original layer was found, and only cracks and a honeycomb appearance were visible, which leads to excluding this bur as an option to obtain a suitable surface roughness.

Furthermore, the findings of this research counter previous studies that achieved great acceptance with the 30-blade tungsten carbide burs [10,15]. We recommend caution with this type of instrument: since it is thinner than the others, small pressure changes in the operator’s hand may cause deep ruts. This high speed method can lead to a greater loss of enamel tissue and increase the furrows effect [24]. The results of the *Y*-axis of surfaces treated with 30-blade tungsten carbide burs showed a predominance of peaks and sharpness as *R*_sk_ and *R*_ku_ values increased. This finding might suggest a protective factor, with less accumulation of plaque. The SEM images of this group were scored as unsatisfactory, according to the increase of grooves and adhesive remnants on the enamel surface. A rougher surface, resulting from a 30-blade tungsten carbide bur, indicates higher bacterial adherence on the surface after procedures using it.

Although an increase of enamel roughness after the removal of carbide burs occurred on the *X*-axis, the inverse was noted on the *Y*-axis, in the same groups. This decrease may be due to different patterns in the enamel roughness. We considered the extrapolation of quantitative assessment compared to the image quality, due to contact of the profilometry feature with a needle-like tip, may lead to a misinterpretation of enamel conditions, as roughness quantification is limited to showing profile topographic changes, as previously demonstrated [25]. In addition, one would expect to find increased initial roughness on the *Y*-axis compared to the *X*-axis due to the cervical-occlusal direction of the enamel prisms’ deposition, but this did not coincide with our findings. Furthermore, the smoothest surface found on the *Y*-axis seemed to represent the similar cutting shape of the carbide burs, already reported [26]. This outcome exhibits a behavioral change of the enamel roughness that is dependent of the removal direction. The same result cannot be found on the diamond bur group, due to its heterogeneous arrangement of diamond particles.

Three height measurements were used to scale the different behaviors of the enamel roughness, not only the *R*_a_ parameter that cannot indicate the depth of the grooves [27,28]. Similar conditions of the related *R*_a_ and *R*_q_ values were expected [8], whereas parameter *R*_z_ showed similarities with the exception of the CVD group in the *Y*-axis. An attempt was made to eliminate the operator’s hand bias by increasing the total sample. Data on the *Y* axis, poorly documented or disclosed, indicates different behavior than the *X* coordinate, with an important analysis option to be used by future profilometry studies using a contact feature. Further research is recommended on bacterial colonization to different roughness parameters and analysis of other parameters such as *R*_Δq_, which presents the angle of the peaks and shows whether the surface causes a reflection of light.

The time required to remove the remnant resin was inversely proportional to the power of the cutting instruments, as occurred in other studies [3,8]. Despite a resemblance among the three groups, Group III demonstrated efficacy compared to the control group. A direct comparison with other studies was not possible due to the different methodologies used. The time required for cleaning with the five-blade carbide was similar to a 2012 study by Ryf et al. [21], which took 65 s removing with an eight-blade carbide bur at slow speed. In addition, previous studies [3,8] found, respectively, that removal required 10.3 s with eight-blade carbide at high speed and 40 s with an eight-blade carbide bur at low speed.

## 4. Materials and Methods

In order to conduct this in vitro study, 65 maxillary premolars extracted for orthodontic reasons were used, following the inclusion criteria which included: complete root development, no carious lesions and cracks in the enamel, evaluated with a stereomicroscope at 20× magnification. The teeth were stored in 0.5% Chloramine T [29] for 1 week. The study was conducted in accordance with the Declaration of Helsinki, and the protocol 0305.0.243.000-11 was approved by the Ethics Committee of the University.

### 4.1. Preparation of Specimens

The teeth were embedded in acrylic resin with the entire crown exposed. The enamel surface was polished with a rubber cup and pumice for 10 s and rinsed with water for 15 s. The bonding area of 25.0 mm^2^ square of the enamel surface was delimitated with a black tape placed in its middle third.

### 4.2. Roughness Test

Sixty teeth were numbered from 01 to 60 and were stored in a glass container with distilled water until the time of the initial roughness recording, and five teeth (healthy group) were used only under the scanning electron microscope (SEM) test. Roughness test was performed with a profilometer Form Talysurf PGI 1000 (Taylor Hobson Ltd., New Star Road 2, Leicester, UK) at the Photonic Lab of the Institute of Advanced Studies (IEAv—São José dos Campos, SP, Brazil). The profilometer tip was perpendicular to the buccal surface of the tooth at a speed of 1 mm/s, cut-off of 0.8 mm and 4.0 mm measurement length in accordance with ISO 3274 [30]. The variations in the length measurement and cut-off were according to the *R*_a_ values obtained at the pilot study. Six analyses were performed per sample, three on horizontal direction, distal to mesial (*X*-axis) and three on vertical direction, gingival to occlusal (*Y*-axis), with each measurement 1.5 mm distance. The following roughness parameters have been identified as a previous study [31]:

- the average roughness (*R*_a_), which is the arithmetic mean of all absolute distances;
- the root mean square of roughness (*R*_q_), which is the distribution of height related to a median line;
- the average height between the five peaks and valleys with the highest and lowest profile points (*R*_z_);
- the flattening of the profile (*R*_ku_), whether the angle of the peaks and valleys is sharp (>3) or obtuse (<3); and
- the symmetry of the profile (*R*_sk_), verifying the predominance of peaks (+) and valleys (−).

### 4.3. Bonding, Adhesive Removal, and Final Roughness Test

The 60 specimens were bonded within the delimitated area to the edgewise brackets (Dental Morelli Ltd., Sorocaba, SP, Brazil) for premolars with a size of 4.00 × 4.00 mm. Each buccal surface of the tooth was etched with 37% orthophosphoric acid for 15 s, rinsed with water and then air-dried for 10 s with a triple syringe. A thin layer of Transbond XT primer was applied on the enamel surface. Transbond XT adhesive resin (3M Unitek Co., Monrovia, CA, USA) was applied at the base of the brackets. Excess adhesive was removed with an explorer, and all samples were light-cured for 30 s with a Radii-Cal LED unit (SDI Ltd., Bayswater, VIC, Australia), with 1250 mW/cm^3^ light-intensity and stored for 24 h in distilled water. Then, all brackets were debonded after squeezing them with Howe pliers. More than 90% of the adhesive remained on the enamel preventing cracks, as previously demonstrated [22]. The enamel surface with no adhesive was on the edges of the bracket area. The samples were then randomized by applying Research Randomizer Form 4.0 software (Social Psychology Network, Middletown, CT, USA) and divided into three groups (*n* = 20), according to the removal method used: Group 1, five-blade tungsten carbide bur—H22GK (Brasseler GmbH & Co., Lemgo, Germany), Group 2, 30-blade tungsten carbide bur—H135UF (Brasseler GmbH & Co., Lemgo, Germany), and Group 3, ultrasonic diamond CVD bur—T0F (CVDentus Ltd.a., São José dos Campos, Brazil). The removal was performed by tungsten carbide burs in high-speed rotation, at 120,000 rpm, and by ultrasonic bur at a 30,000 Hz frequency, all with water cooling. The removal occurred in the direction of the blades and involved a single direction with a dental loupe with a 1.5× magnification. The tungsten carbide burs were exchanged after the removal of 10 samples. After removal, the samples were yet again evaluated by profilometry analysis and 3D images of 1.00 × 1.00 mm were obtained. Bonding and debonding procedures were performed by a single operator (F.D.). The time of each adhesive removal was recorded in seconds. The recordings started after the first contact between the bur and adhesive and stopped after all visible adhesive was removed by the operator. Five specimens of the three removal groups were randomly selected for SEM image analysis.

### 4.4. Micromorphological Analysis

SEM analyses were performed by a JSM-6360 microscope (JEOL Ltd., Tokyo, Japan) with 10 kV. Imaging by secondary electrons of 20× magnification were obtained and evaluated by one blinded and calibrated examiner (V.F.). The examiner calibration occurred with a photomicrograph template used before [32] at two different times with an interval of seven days. Five different images of each group were printed on photographic paper and classified into four groups, according to the Enamel Damage Index (EDI) of Shuler and van Waes [32]: score 0—perfect surface (minimal grooves and presence of the layer), score 1—satisfactory surface (little adhesive remnants on enamel areas with and without slots and the presence of an enamel layer), score 2—imperfect surface (presence of grooves and rough adhesive remnants, with or without enamel layer) and score 3—unacceptable surface (cracks in the enamel, with or without adhesive remnants). The results reflect the highest scores amount, at a total of 10 analyses per group (*n* = 5), which were obtained during the experimentation phase.

### 4.5. Satistical Analysis

Sample calculation was performed by G*Power 3.1.3 (Heinrich Heine Universität, Düsseldorf, Germany) with a power study of 80% and *α* = 0.05, totaling a minimum number of 18 specimens per group, in accordance with a previous pilot study. Data normality was checked applying the Shapiro-Wilk test for measures distribution. Within each group, differences between the initial and final surface roughness were analyzed, and the Wilcoxon signed rank test was used to pair the differences. A roughness analysis among groups and the time required for adhesive removal were performed with the Kruskal-Wallis and the Dunn’s multiple comparison test. The examiner calibration was performed by kappa coefficient analysis (*k* = 0.88). All statistical tests were calculated using STATA 12.0 for Windows (StataCorp LP, College Station, TX, USA). Significance for roughness tests was adopted with probability values *p* < 0.001 and for time removal *p* < 0.05.

## 5. Conclusions

Enamel roughness has changed after removal of the orthodontic adhesive remnants. Carbide and CVD diamond burs were not successful in restoring the original condition of enamel roughness; although, the five-blade resulted in less enamel roughness than the 30-blade and CVD burs. The CVD bur removed adhesive faster than carbide burs. Removing adhesive remnants with 30-blade and CVD burs resulted an undesirable enamel surface.

## Figures and Tables

**Table 1:** Results of the Mean and Standard Deviation (SD) of the Surface Roughness within Groups (*X*-axis), in µm.

Parameter	Group	Initial Mean ± SD	*p* Value	Final Mean ± SD	*p* Value	Dunn ^††^
*R*_a_	1	2.09 ± 0.75	0.1563	2.73 ± 1.08	<0.001	A
	2	2.01 ± 0.78	-	3.36 ± 1.46 ^†^	-	B
	3	2.43 ± 0.61	-	3.82 ± 0.62 ^†^	-	C
*R*_q_	1	2.57 ± 0.89	0.0989	3.51 ± 1.53	<0.001	A
	2	2.55 ± 1.01	-	4.34 ± 1.94 ^†^	-	B
	3	3.05 ± 0.81	-	4.79 ± 0.78 ^†^	-	C
*R*_z_	1	7.35 ± 2.47	0.0611	11.28 ± 5.47 ^†^	<0.001	A
	2	7.32 ± 3.41	-	13.04 ± 5.51 ^†^	-	A
	3	7.90 ± 2.33	-	17.99 ± 3.02 ^†^	-	B
*R*_ku_	1	2.96 ± 1.23	0.3734	3.40 ± 1.38	0.3965	-
	2	3.26 ± 1.51	-	3.56 ± 1.49	-	-
	3	3.01 ± 1.12	-	3.06 ± 0.73	-	-
*R*_sk_	1	−0.22 ± 0.63	0.1556	−0.32 ± 0.58	0.1354	-
	2	−0.43 ± 0.70	-	−0.32 ± 0.67	-	-
	3	−0.34 ± 0.66	-	−0.16 ± 0.42	-	-

^†^ Significant difference between Initial and Final Roughness Means (*p* < 0.001). ^††^ No statistically significant differences with the same letters (*p* < 0.001).

**Table 2:** Results of the Initial and Final Roughness Means and Standard Deviation (SD) within Groups (*Y*-axis), in µm.

Roughness Parameter	Group	Initial Mean ± SD	*p* Value	Final Mean ± SD	*p* Value	Dunn ^††^
*R*_a_	1	2.04 ± 0.60	0.0610	1.21 ± 0.59 ^†^	<0.001	A
	2	1.80 ± 0.62		0.91 ± 0.28 ^†^		A
	3	2.04 ± 0.54		3.08 ± 0.72 ^†^		B
*R*_q_	1	2.58 ± 0.78	0.0568	1.73 ± 0.94 ^†^	<0.001	A
	2	2.27 ± 0.84		1.23 ± 0.44 ^†^		B
	3	2.57 ± 0.70		3.95 ± 0.91 ^†^		C
*R*_z_	1	8.58 ± 2.79	0.0832	6.66 ± 3.32 ^†^	<0.001	A
	2	7.46 ± 2.75		4.58 ± 1.56 ^†^		B
	3	8.64 ± 2.36		17.19 ± 3.40 ^†^		C
*R*_ku_	1	3.67 ± 3.33	0.8477	5.59 ± 2.41 ^†^	<0.001	A
	2	2.94 ± 0.81		5.34 ± 2.73 ^†^		A
	3	3.05 ± 1.19		3.51 ± 0.93		B
*R*_sk_	1	0.09 ± 0.90	0.0558	−0.13 ± 0.94	<0.001	A
	2	0.12 ± 0.47		0.80 ± 1.02 ^†^		B
	3	−0.34 ± 0.49		−0.31 ± 0.39		C

^†^ Significant difference between Initial and Final Roughness Means (*p* < 0.001). ^††^ No statistically significant differences with the same letters (*p* < 0.001).

**Table 3:** Evaluation of SEM images.

Groups	Score 0	Score 1	Score 2	Score 3
Healthy	07	03	-	-
5-blade	03	05	02	-
30-blade	-	04	06	-
CVD	-	-	01	09

**Table 4:** Time required (in seconds) to remove adhesive remnant.

Group	Mean (±SD) ^a^	Minimum	Maximum
5-bladed	75.5 (±29.6) A	33	136
30-bladed	59.2 (±17.8) AB	32	93
CVD	54.8 (±22.9) B	28	114

^a^ Groups with the same letter are not different (*p* < 0.05).
